# Environmental Enteric Dysfunction: Reemergence of an Old Disease

**DOI:** 10.1093/infdis/jiab454

**Published:** 2021-09-22

**Authors:** Peter B Sullivan

**Affiliations:** Department of Paediatrics, University of Oxford, Oxford University Hospitals NHS Foundation Trust, Oxford, United Kingdom

**Keywords:** environmental enteropathy, environmental enteric dysfunction, tropical enteropathy

There is no universally agreed definition of environmental enteric dysfunction (EED) but is it generally regarded to be an acquired subclinical disorder of the small intestine, characterized by villous atrophy, crypt hyperplasia, and lymphocytic infiltration of the lamina propria [1]. This state of chronic intestinal inflammation is associated with reduced absorptive capacity, and reduced barrier function in the small intestine, and low-grade systemic inflammation. EED has been proposed to underlie stunted growth among children in lower- and middle-income countries and is currently the subject of intense research interest.

Description of a clinical condition similar to EED has a long history. It had been known since the discovery and subsequent colonization of the New World in the 15th and 16th centuries that some of the Europeans who came to live on the tropical islands of the Americas were afflicted with a new disease causing weight loss and diarrhea. The first English language description of a such a disorder was in the mid-18th century by William Hillary who described malabsorption in European expatriates in Barbados [2]. More than 100 years later, Patrick Manson, the father of tropical medicine, described a similar disorder in expatriates in the Dutch West Indies, and he used the term tropical “sprouw,” from a Dutch word meaning stomatitis [3].

This disease, subsequently known as “tropical sprue,” was soon recognized in established European communities in South Asia. Tropical sprue is a primary malabsorption syndrome that runs a chronic relapsing course. It is characterized by a sore tongue, diarrhea with steatorrhea, progressive weight loss, and cachexia. It is associated with a macrocytic anemia and megaloblastic bone marrow. In addition to occurring in previously healthy expatriates residing in endemic areas for more than a year, reports of epidemic malabsorption in British and American troops in South Asia during the Second World War were also common and gave rise to the concept of gut lesions caused by environmental, and likely infectious, factors [4–6].

It appeared that this sprue occurred primarily in those tropical areas with a high rainfall, and that it affects the indigenous populations as well as Caucasians, Chinese, and other immigrants. It is relatively rare throughout Africa, although there are cases reported from Nigeria, Central and South Africa, Uganda, and the Sudan. Tropical sprue is seen in adults of both sexes much more often than in children.

When biopsy of the small intestine became possible and was applied to cases of tropical sprue it was seen that abnormalities of small-bowel structure and function were an integral part of the pathology. Histological abnormalities were often accompanied by excess fecal fat excretion and malabsorption of xylose and vitamin B_12_. Further study of intestinal morphology by jejunal biopsy in asymptomatic individuals from Africa, Asia, and Latin America found common abnormalities of shorter and thickened villi, increased crypt depth, and inflammatory cellular infiltration, which did not necessarily result in overt symptoms. Studies in India by Marsh and colleagues indicated that lymphocyte activation, suggestive of a local cell-mediated immune reaction, occurred in tropical sprue but was secondary to damage already inflicted on enterocytes [7].

In 1966, a seminal study by Lindenbaum found that 40% of Peace Corps volunteers stationed in (East) Pakistan had signs of malabsorption and none of their jejunal biopsies had normal finger-like villous architecture but showed varying degrees of abnormality [8]. Lindenbaum and colleagues went on to show that the small-intestinal disorder acquired by these volunteers who had lived in the tropics appeared to be reversible on their return to the United States and to have no permanent sequelae in most subjects [9, 10].

Observers began to conclude that the majority of inhabitants of tropical countries probably had subclinical small-intestinal disease [11–13]. The mucosal changes in tropical sprue as well as in asymptomatic subjects are nonspecific and represent the typical response of the small-bowel mucosa to a variety of noxious stimuli. The asymptomatic abnormalities of the small intestine became known as “tropical enteropathy.”

The initial descriptions of tropical enteropathy did not define the true extent of the problem. Initial reports focused on adults and the realization that children were affected by the same disorder was delayed. Moreover, while various causes of this intestinal dysfunction have been postulated, the definitive cause has yet to be determined [14]. Tropical enteropathy was shown to be associated with mucosal T-cell activation and crypt hyperplasia [15].

The nomenclature used to describe this asymptomatic enteropathy has evolved in response to ongoing efforts to better understand and characterize the disease. Tropical enteropathy was renamed “environmental enteropathy” in the late 2000s in recognition of emerging evidence that the quality of the environment was more important than climate or latitude; the condition was not limited to tropical areas, nor does it affect all residents in the tropics. More recently, it has been further renamed EED to better capture the functional, as well as structural, abnormalities associated with this condition [16].

The mechanisms through which environmental conditions predispose to enteric dysfunction are increasingly being elucidated with evidence of enteric infection and intestinal dysbiosis producing abnormalities in intestinal permeability [17, 18]. Biomarkers of intestinal barrier disruption by epithelial inflammation have helped to understand more about the pathophysiology of EED and its growth and developmental consequences in children [19, 20]. The effects of intestinal and systemic inflammation that are integral to the pathophysiology of EED contribute to low nutrient absorption and anemia, leading to altered brain development in young children and subsequent poor neurocognitive development [21]. Although the mechanisms through which EED is associated with poor responses to oral vaccine are not well understood, it is again likely that intestinal and systemic inflammation are the major causes [20, 22, 23].

The realization that such subclinical gut disease is probably the main cause of childhood stunting, and there are over 150 million growth-stunted children younger than 5 years worldwide, as well as low efficacy of oral vaccines and poor neurocognitive development, has led to an explosion of research into EED in the last 2 decades.

The exponential increase in research (and grant funding) in the field of environmental enteropathy has accomplished a great deal in terms of elucidating the underlying mechanisms of the complex interactions taking place at the small-intestinal luminal-mucosal interface. Moreover, there will be more to come from the application of the latest technologies including proteomics, transcriptomics, and application of machine learning techniques to mucosal assessment.

## Notes

***Potential conflicts of interest.*** The author certifies no potential conflicts of interest. The author has submitted the ICMJE Form for Disclosure of Potential Conflicts of Interest. Conflicts that the editors consider relevant to the content of the manuscript have been disclosed.
